# CXCL1/CXCR2 is involved in white matter injury in neonatal rats via the gut–brain axis

**DOI:** 10.1186/s12868-022-00749-1

**Published:** 2022-11-19

**Authors:** Can Yang, Zhiyuan Feng, Hong Deng, Lu Dai, Ling He, Linlin Yin, Jing Zhao

**Affiliations:** Department of Neonatology, Affiliated Hospital of North Sichuan Medical College, No. 1 Maoyuan South Road, Shunqing District, Nanchong, 637000 Sichuan China

**Keywords:** White matter injury, Necrotizing enterocolitis, Gut–Brain axis, CXCL1, CXCR2

## Abstract

**Background:**

This study aimed to investigate whether CXCL1/CXCR2 mediates intestinal injury or white matter injury by delivering inflammatory mediators through the gut–brain regulation axis.

**Methods:**

Neonatal SD rats, regardless of sex, were administered 3% dextran sulfate sodium via intragastric administration at different time points to construct necrotizing enterocolitis (NEC) models. Meanwhile, hypoxia and ischemia were induced in 3 day-old SD rats to construct hypoxic–ischemic brain injury (HIBI) and NEC + HIBI models, without gender discrimination. Hematoxylin–eosin staining was used to observe pathological changes in neonatal rat intestinal and brain tissues. Western blotting detected CXCL1 and CXCR2 expression in NEC, HIBI, and NEC + HIBI rat intestinal and brain tissues.

**Results:**

Compared with normal rats, pathological damage to periventricular white matter was observed in the NEC group. In addition to the increased mortality, the histopathological scores also indicated significant increases in brain and intestinal tissue damage in both HIBI and NEC + HIBI rats. Western blotting results suggested that CXCL1 and CXCR2 expression levels were upregulated to varying degrees in the intestinal and brain tissues of NEC, HIBI, and NEC + HIBI neonatal rats compared to that in the normal group. Compared with the HIBI group, the expression of CXCL1 and CXCR2 continued to increase in NEC + HIBI rats at different time points.

**Conclusions:**

CXCL1/CXCR2 may be involved in white matter injury in neonatal rats by delivering intestinal inflammatory mediators through the gut–brain axis.

**Supplementary Information:**

The online version contains supplementary material available at 10.1186/s12868-022-00749-1.

## Highlights

1. Pathologically impaired periventricular white matter was observed in NEC neonatal rats.
2. Hypoxic-ischemic brain injury can also lead to intestinal inflammation.
3. CXCL1 and CXCR2 were significantly upregulated in intestinal and brain tissues in NEC, HIBI, and NEC+HIBI rats compared to the normal group.
4. Compared with the HIBI group, CXCL1 and CXCR2 expression continued to increase in NEC+HIBI rats.

**Supplementary Information:**

The online version contains supplementary material available at 10.1186/s12868-022-00749-1.

## Background

Statistically, approximately 15 million premature babies are born worldwide each year. With an 11% probability of occurrence, there is an inevitable risk of brain damage in these preterm births, despite breakthroughs in modern perinatal medicine [1]. Perinatal brain injury refers to cerebral ischemia and/or hemorrhagic damage in premature infants at the critical stage of development due to hypoxic–ischemic encephalopathy, inflammation, infection, and other pathological factors, which can also lead to long-term sequelae of the nervous system or even death in severe cases [2]. In an epidemiological survey of 3378 premature infants, 798 (23.56%) were diagnosed with brain injury [3]. White matter injury is a unique form of brain injury in preterm infants, and studies have found that preterm infants with periventricular white matter malacia are at an increased risk of cerebral palsy [4, 5]. Therefore, brain injury in premature infants has become a serious public health problem, leading to motor impairment, cognitive impairment, and audio-visual impairment. Necrotizing enterocolitis (NEC) can cause acute intestinal necrosis in premature infants and can potentially cause severe neurological impairment and increase the risk of brain damage [6–8]. Epidemiological studies revealed that 45–61% of patients with neonatal NEC are neurologically impaired, and infants with NEC had a 1.6-fold increased risk of developing neurodevelopmental disorders compared with peers without NEC [9, 10]. Likewise, intestinal inflammation during traumatic brain injury can lead to sustained systemic immune responses and changes in autonomic nerve balance through the gut–brain axis [11]. Feng et al. found that chronic colitis may promote T-cell migration from the intestinal tract to the meninges and influence the M1 and M2 microglia/macrophage imbalance, exacerbating ischemic brain injury in mice [12]. Based on these findings, we hypothesized that neonatal brain injury may be associated with NEC. However, the underlying mechanism of the gut-brain regulation axis has not been clarified.

The chemokine family and their receptors control the migration and residence of immune cells and are closely related to inflammatory responses [13]. Moreover, hypoxic–ischemic and inflammatory responses are key factors affecting premature brain injury [14]. As a member of the chemokine family, C–X–C motif chemokine ligand 1 (CXCL1) binds to its specific receptor, C–X–C motif chemokine receptor 2 (CXCR2), resulting in chemotaxis of T-cells, monocytes, and neutrophils in the brain [15]. CXCL1 has also been shown to play a critical role in producing reactive oxygen species, which consequently regulate further inflammation [16]. Furthermore, the CXCL1/CXCR2 pathway may be involved in inflammation–induced hypoxic-ischemic brain injury (HIBI) in neonatal rats and pro-inflammatory microglial activation [17]. Yellowhair et al. illustrated that increased CXCL1/CXCR2 signaling in the brain could stimulate immune cell activation and neutrophil recruitment [18]. CXCL1 was also involved in pathways of reactive oxygen species generation during neutrophil recruitment in the circulating blood of patients with NEC [19]. Downregulated activation of CXCR2-related pathways can improve intestinal injury and inflammatory responses in newborn NEC mice [20]. Based on the above studies, we speculated that the expression of CXCL1/CXCR2 may promote inflammatory responses in NEC and brain injury. However, whether CXCL1/CXCR2 affects neonatal white matter injury by transmitting intestinal inflammation through the gut-brain regulatory axis has not been yet reported.

Therefore, this study aims to explore the relationship between neonatal brain injury and NEC, and to determine whether CXCL1/CXCR2 can transmit intestinal inflammation and affect white matter injury through the gut-brain regulatory axis. For this purpose, this study first induced NEC in neonatal rats to detect pathological changes in periventricular white matter, as well CXCL1 and CXCR2 expression changes in intestinal and brain tissues. Considering that 3–7 day-old neonatal rats may simulate the highest risk period of preterm brain injury (23–26 weeks of gestation) [4, 21], and the preference for white matter injury may decline rapidly after birth [22], we selected 3 day-old SD neonatal rats to construct HIBI and NEC + HIBI rat models and to observe behavioral and pathological changes and CXCL1 and CXCR2 expression changes in intestinal and brain tissues. Our study proposes potential therapeutic targets for neonatal white matter injury and provides a theoretical basis for further understanding the mechanism of the gut–brain regulation axis in premature brain injury.

## Materials and methods

### Animals

Parturient Sprague Dawley (SD) rats and their newborns were provided by the Experimental Animal Center of North Sichuan Medical College (License No: SYSK [Sichuan] 2018-076). Maternal rats were bred professionally under specific pathogen-free conditions until natural delivery. Newborn rats were placed in the same cage with their mothers and fed breast milk freely. A total of 43 newborn 1 day-old SD rats were used to construct the NEC model, without gender discrimination. Additionally, 60 3 day-old SD rats weighing 5–7 g each were selected for HIBI model construction regardless of sex.

### Experimental NEC model

Forty-three newborn SD rats were randomly divided into 21 experimental cases and 22 controls. NEC was induced with 3% dextran sulfate sodium salt (DSS) dissolved in normal saline. Each NEC rat was administered 0.1 mL intragastric gavage at an interval of 6 h, 4 times a day for 3 days. Rats in the control group were administered the same dose of normal saline at the same time. At 3, 24 and 72 h after gavage, neonatal SD rats were sacrificed by cervical dislocation (6 rats in each group at each time point), and the whole brain and intestine tissues were collected for western blot (WB) experiments to detect the expression levels of target proteins. Furthermore, at 72 h time point, pathological sections of brain and intestinal tissues were taken from 3 rats in each group for pathological tissue detection. Based on the histologic injury score of NEC defined by Ginzel et al. (Additional file 5: Table S1), neonatal rats with intestinal histopathological scores ≥ 2 were considered NEC-positive [23].

### HIBI and NEC + HIBI model construction and experimental grouping

Sixty 3 day–old SD rats were assigned for HIBI and NEC + HIBI model construction (17 rats in normal, 20 rats in HIBI, and 23 rats in NEC + HIBI groups). Rats were anesthetized with isoflurane, and the right common carotid artery and permanent ligation of the proximal and distal ends were separated using silk thread for ischemia treatment. The rats were then placed in an oxygen-deficient chamber (containing 8% oxygen and 92% nitrogen) 30 min after ligation for 3 h for hypoxia treatment. The NEC + HIBI group was given 3% DSS by gavage 4 times a day for 3 consecutive days from birth, and hypoxia–ischemia treatment was administered on the third day. Neonatal rats in the HIBI group received normal saline via intragastric administration at the same time after birth, followed by hypoxia–ischemia treatment. Rats in the control group were given normal saline via intragastric administration after birth, and the right common carotid artery was separated without ligation or hypoxia treatment. Sampling and analysis were performed at 3, 12, 24, and 72 h after model construction. The success of the animal model was determined by the behavioral changes, and pathological features of brain and intestinal tissues. General behavioral observations included mortality, changes in milk intake, abdominal distension and diarrhea, changes in body weight and skin color, and convulsions. Intestinal injury and NEC positivity were determined by the scoring criteria in Additional file 5: Table S1. Pathological changes in brain tissue were identified according to the grading method (Additional file 5: Table S2) by Uehara et al. [24].

During the treatment, 2 rats in normal, 5 rats in HIBI, and 8 rats in NEC + HIBI groups died. Finally, a total of 45 rats (15 rats in each group) were included in the subsequent analysis. Rats in each group were anesthetized and sacrificed by cervical dislocation at 3, 12, 24 and 72 h after treatment, and the intestinal and brain tissues were harvested. At time points of 3, 12 and 24 h, 3 rats in each group were sacrificed for WB experiments; while 6 rats were sacrificed at the 72 h time point, of which 3 were used for WB experiments and the other 3 were used for pathological examination.

### Sample collection

All SD rats included in the analysis were anesthetized with sevoflurane and sacrificed by cervical dislocation. The scalp was cut in the middle along the top of the head to the nose, and the skull was pushed aside, leaving the brain exposed. The whole brain tissue was separated from the optic nerve and brainstem and then placed in a specimen box. For intestinal tissue collection, we first opened the abdominal cavity from the ventral midline and rapidly separated the mesentery and blood vessels. Then, the antral and duodenal junction, mesenteric reticulum, and rectum near anus were identified and quickly cut, followed by the removal of the entire intestinal tissue. The intestine tissue was washed with sterile saline at 4 °C and also placed in a specimen box. All specimen boxes were frozen in liquid nitrogen for 10 min, and then stored in − 80 °C refrigerator until used in paraffin sectioning.

### Hematoxylin and eosin (H&E) staining

The intestinal and brain tissues of NEC, HIBI, and NEC + HIBI rats 72 h after treatment were histopathologically observed using H&E staining. The preserved tissue samples were prepared as paraffin sections after fixation, dehydration, embedding, and sectioning. Dewaxed paraffin sections received the hematoxylin solution and were incubated for 5 min at room temperature. After washing and differentiation for 30 s, paraffin sections were treated with eosin for 2 min, followed by xylene decolorization and neutral gum sealing. Finally, a light microscope (400 ×) was used to observe the pathological features of the ileocecal and periventricular tissues.

### WB analysis

We used WB to detect CXCL1 and CXCR2 expression at the protein level in neonatal rats’ intestinal and brain tissues under different treatment conditions. Total protein was extracted using cell lysis buffer (P0013, Beyotime) and quantified using a BCA Protein Assay Kit (P0009, Beyotime). We used a polyacrylamide gel electrophoresis (PAGE) preparation kit (PG112, EpiZyme) to perform sodium dodecyl sulfate (SDS)-PAGE, before transferring the proteins to an Immobilon-PSQ PVDF membrane (ISEQ00010, Sigma-Aldrich). The membranes were incubated with anti-CXCL1 (1:1000, AF5403, Abcam), anti-CXCR2 (1:1000, DF7095, Abcam), and anti-β-actin (1:100,000, AC026, ABclonal) antibodies.β-actin was used as an internal reference. After incubation with the secondary antibody (1:5000, goat anti-rabbit IgG (H + L), ab6721, Abcam), the membranes were treated using an ECL luminescence kit (KF001, Affinity) and visualized using a Universal Hood II gel imager (Bio-Rad). A detailed protocol for western blotting analysis can also be found in Kurien et al. [25].

### Statistical analysis

Statistical analysis was performed using SPSS 23.0, and all data are presented as the mean ± standard deviation in this study. Comparisons between multiple groups were performed using a one-way ANOVA. The least significant difference test was used for homogeneity of variance, and Tamhane’s T2 test was used for heterogeneity of variance. Statistical significance was set at *P* < 0.05.

## Results

### Pathological manifestations of intestinal and brain tissues in NEC neonatal rats

**Fig. 1:**
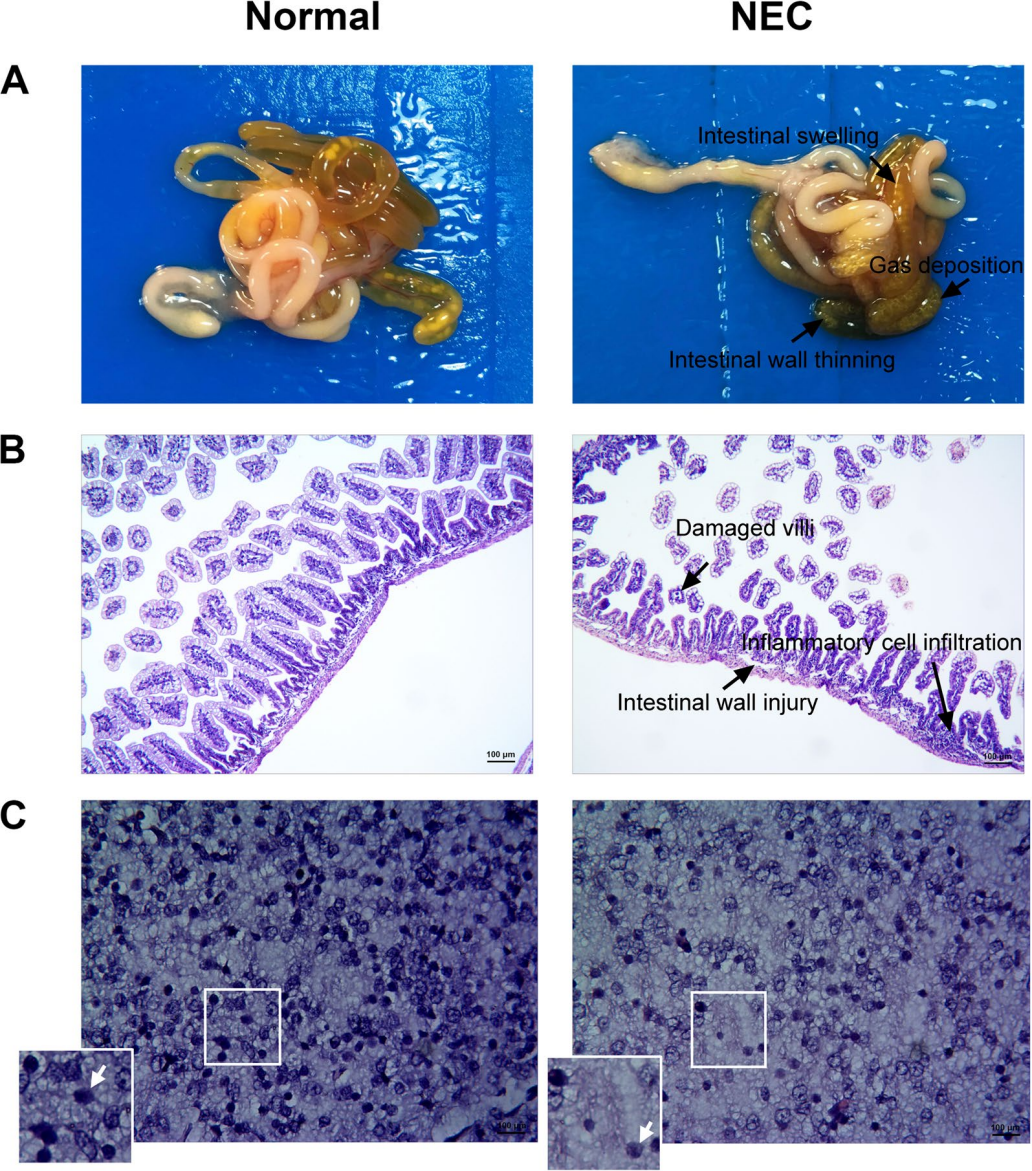
Pathological intestine and brain changes in NEC neonatal rats 72 h after intragastric administration (3 rats for each group). **A** Pathological intestinal tissue features in the control group and NEC neonatal rats. **B** H&E staining results suggest that NEC neonatal rats displayed pathological intestinal tissue changes, with pathological scores greater than 2, accompanied by inflammatory cell infiltration. **C** Pathological changes in the periventricular white matter of NEC neonatal rats observed using H&E staining. The arrow points at the nerve cells

Compared with the control group, intestinal tissues of newborn NEC rats showed intestinal swelling, apparent intestinal gas deposition, and intestinal wall thinning 72 h after intragastric administration (Fig. 1A). H&E staining suggested that the intestinal tissue structure of the control group was intact, but the villi of the NEC group were damaged, accompanied by a certain number of inflammatory cell infiltrations (Fig. 1B). The histological score of intestinal tissues revealed a significant difference (*P* = 0.016) between normal (0 ± 0) and NEC (2.33 ± 0.58) groups (Table 1), which further indicted a successful construction of NEC models. Furthermore, compared to brain tissue of the control group, the NEC group had unclear tissue hierarchy, loose periventricular white matter, and reduced glial cells 72 h after intragastric administration (Fig. 1C). The quantitative results of white matter damage (Table 1) showed that the score of NEC group was 1.17 ± 0.29, which was significantly higher than that of normal group (*P* = 0.016). These results indicated that the degree of brain injury in NEC rats was significantly higher compared with normal rats, and also suggested that NEC may cause several pathological changes in brain tissues.

**Table 1:** Comparisons of intestinal and brain injury scores between normal and NEC groups

Group	Intestinal injury score	Brain injury score
Normal (n = 3)	0 ± 0	0 ± 0
NEC (n = 3)	2.33 ± 0.58	1.17 ± 0.29
F	16	16
*P*	0.016*	0.016*

*NEC* necrotizing enterocolitis

^*^P < 0.05 indicates statistical significance

### CXCL1 and CXCR2 expression is upregulated in the intestines and brains of NEC rats

**Fig. 2:**
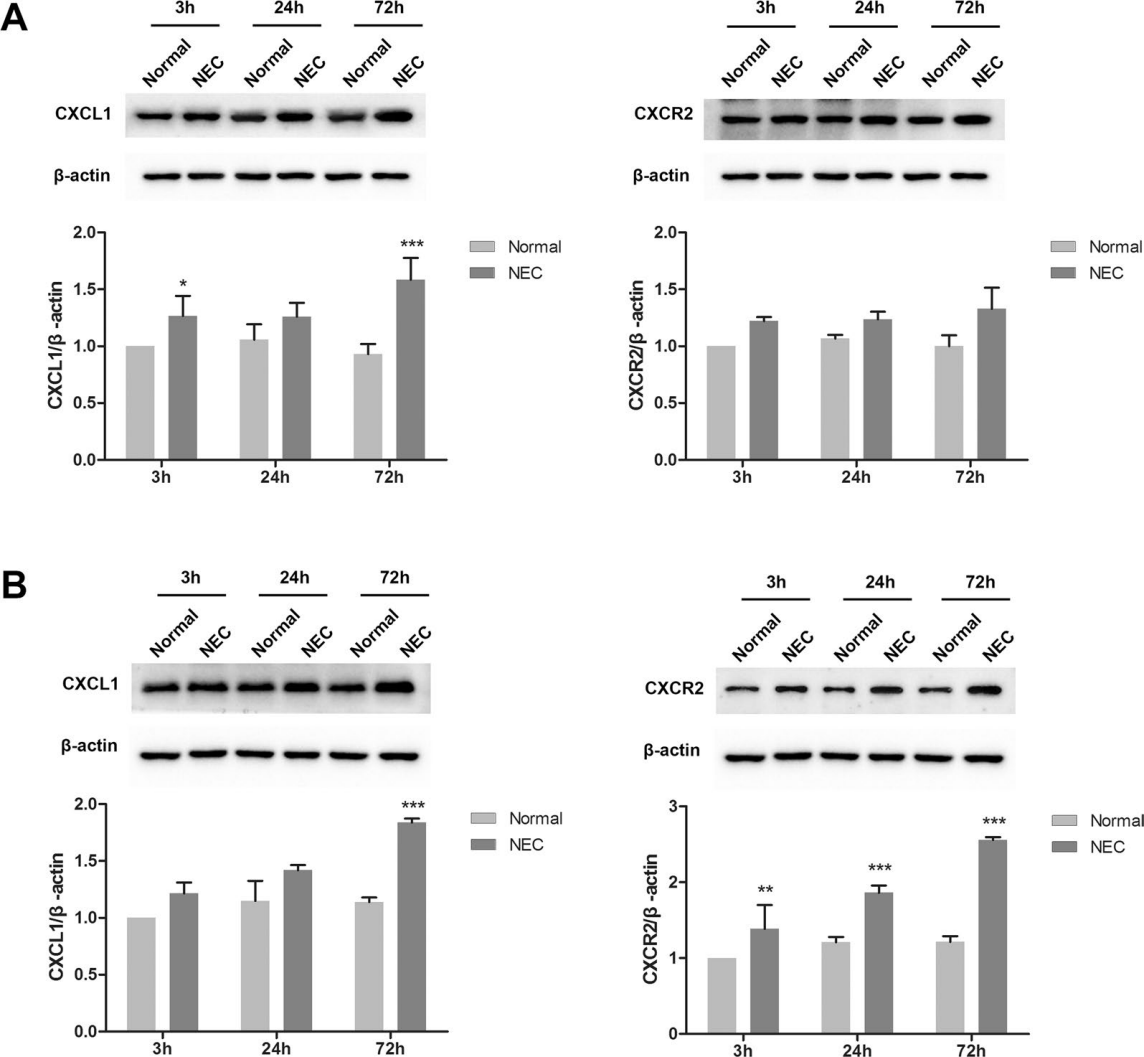
CXCL1 and CXCR2 expression is upregulated in the intestine (**A**) and brain (**B**) tissues of NEC rats compared with the control group 3, 24, and 72 h after intragastric administration (six rats in each group at each time point). *P < 0.05, **P < 0.01, and ***P < 0.001 compared with the normal group

Using western blotting, we further detected CXCL1 and CXCR2 expression differences in the brain and intestinal tissues of control and NEC rats 3, 24, and 72 h after intragastric administration. The results suggested that in the intestinal tissues (Fig. 2A), the relative expression of CXCL1 was significantly increased in NEC rats 3 (1.27 ± 0.18 vs. 1) and 72 (1.58 ± 0.19 vs. 0.93 ± 0.09) h after intragastric administration compared to the control groups (*P* < 0.05). There was no significant difference in CXCR2 expression between the two groups at different gavage times. Furthermore, in brain tissues (Fig. 2B), CXCL1 was significantly upregulated in NEC rats 72 h (1.84 ± 0.04 vs. 1.14 ± 0.04) after intragastric administration compared to the control group (*P* < 0.001). The relative expression of CXCR2 was also notably upregulated in the NEC group 3 (1.38 ± 0.32 vs. 1), 24 (1.86 ± 0.10 vs. 1.21 ± 0.07), and 72 (2.56 ± 0.03 vs. 1.21 ± 0.08) h after intragastric administration, compared to their control groups (*P* < 0.01). These results indicated that damage to the white matter and intestine in NEC rats may promote the increased expression of CXCL1 and CXCR2 in these animals. Raw data of Fig. 2 including three replications are shown in Additional file 1: Figure S1 and Additional file 2: Figure S2.

### Behavioral and pathological features of HIBI and NEC + HIBI rats

**Fig. 3:**
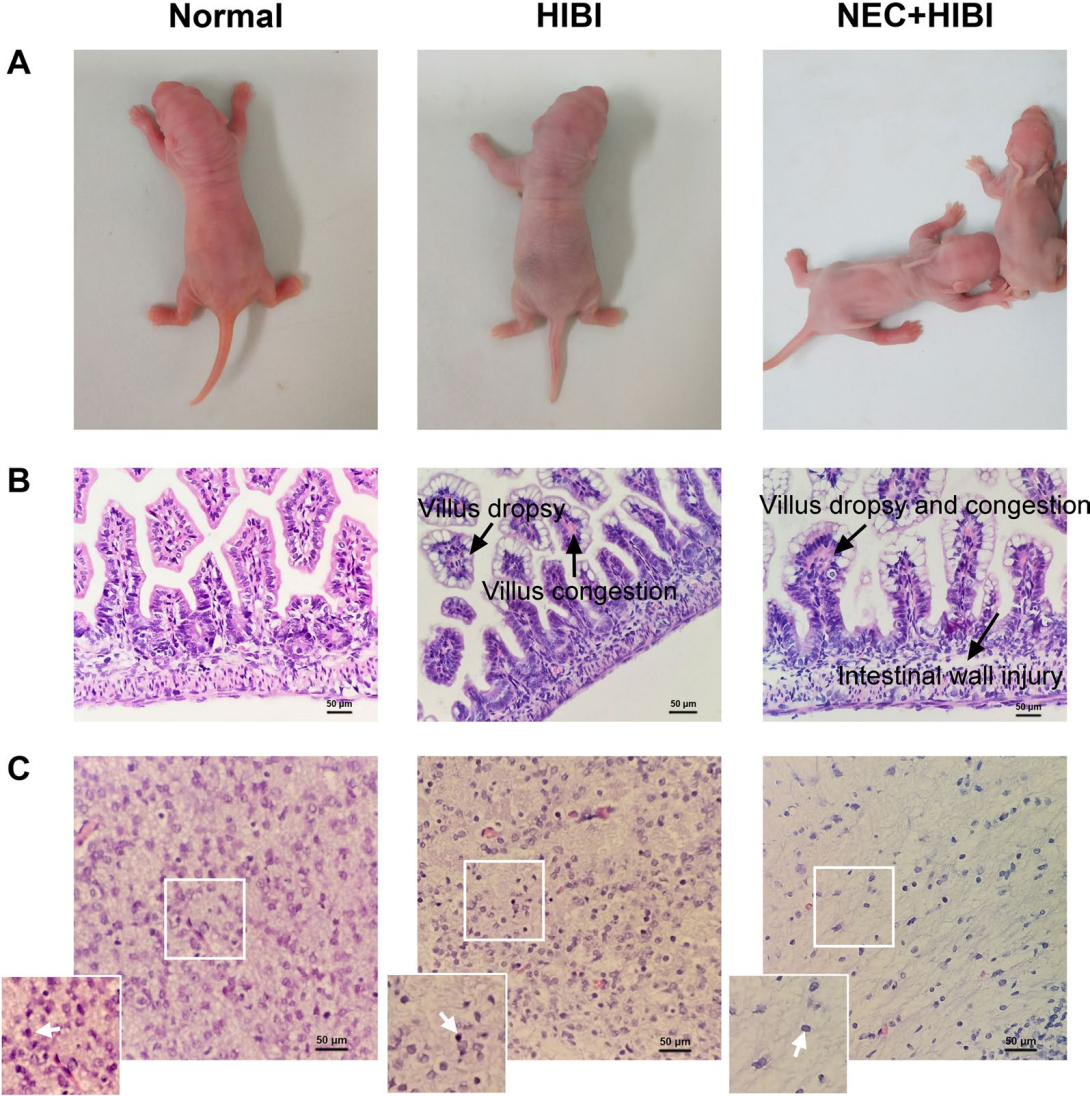
Pathological intestine and brain tissue changes in HIBI and NEC + HIBI rats. **A** Behavioral manifestations of HIBI (20 rats in total with five deaths) and NEC + HIBI in neonatal rats (23 rats in total with eight deaths) compared to the normal group (17 rats in total with two deaths). (**B**, **C**): Pathological changes in the intestinal (**B**) and brain (**C**) tissues observed under a 400 × microscope 72 h after intragastric administration (3 rats in each group). The arrow points at the nerve cells

In terms of behavior and state, neonatal rats in the HIBI group showed cyanosis, limb twitching, abdominal distension, diarrhea, reduced activity, and growth retardation, with a mortality rate of 25% (Fig. 3A). Furthermore, neonatal rats in the NEC + HIBI group had a higher mortality rate of 34.78% compared with HIBI rats and showed behavioral changes, such as pale skin, bloody stools, and growth restrictions (Fig. 3A). Compared with the control group 72 h after intragastric administration, the intestinal tissues of the HIBI group showed moderate villus dropsy and congestion, while the intestinal tissues of the NEC + HIBI group showed severe intestinal wall injury and villus dropsy and congestions (Fig. 3B). These quantitative scores of intestinal tissue characteristics (Table 2) suggest that the degree of intestinal injury in HIBI (1.17 ± 0.29) was significantly higher than that in normal group (*P* < 0.05), while the malignant degree of intestinal tissues in NEC + HIBI group (3.33 ± 0.58) was also significantly increased compared with HIBI rats (*P* < 0.01). We also found that the white matter fibers around the right ventricle were loose and disordered in the HIBI group. Brain tissues in the NEC + HIBI group showed loose and irregularly arranged fibrous structures, reduced nerve cells, and formed mesh-like softening foci (Fig. 3C). To be quantified (Table 2), the brain injury score increased in normal, HIBI (1.17 ± 0.29), and NEC + HIBI (1.83 ± 0.29) groups, indicating that the HIBI model was successfully constructed and the degree of brain injury was significantly aggravated (*P* < 0.05).

**Table 2:** Comparisons of intestinal and brain injury scores between normal, HIBI, NEC + HIBI groups

Group	Intestinal injury score	Brain injury score
Normal (n = 3)	0 ± 0	0.17 ± 0.29
HIBI (n = 3)	1.17 ± 0.29^*^	1.17 ± 0.29^*^
NEC + HIBI (n = 3)	3.33 ± 0.58^##^	1.83 ± 0.29^#^
F	61.8	25.33
*P*	< 0.001	0.001

*HIBI* hypoxic-ischemic brain injury, *NEC* necrotizing enterocolitis

^*^Compared with normal; ^#^compared with HIBI

^*^P < 0.05, ^#^*P* < 0.05, ^##^*P* < 0.01 indicate statistical significances

### CXCL1 and CXCR2 expression is upregulated in HIBI neonatal rats via the gut–brain axis

**Fig. 4:**
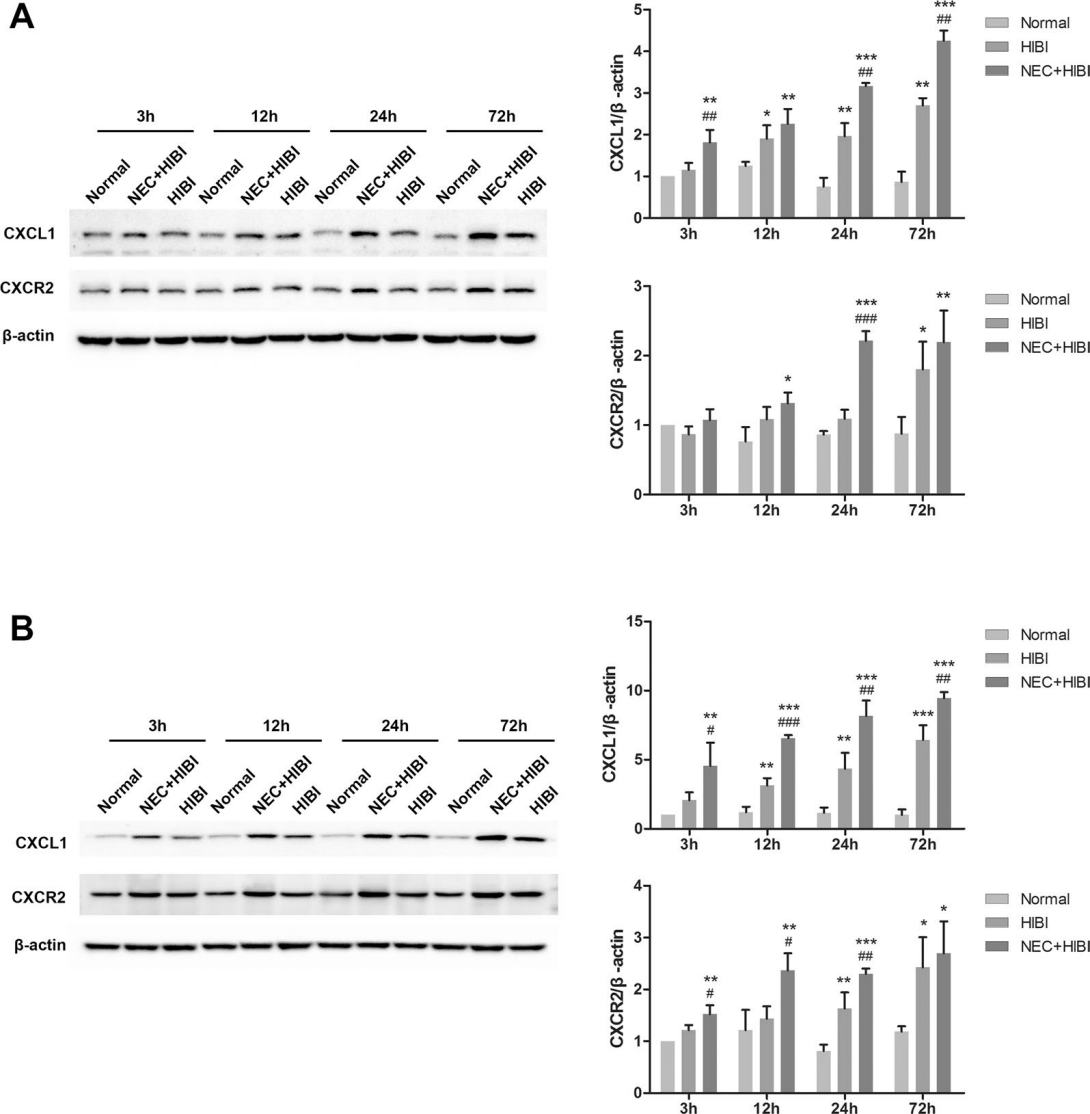
CXCL1 and CXCR2 expression changes in intestinal (**A**) and brain (**B**) tissues of HIBI and NEC + HIBI neonatal rats (3 rats in each group at each time point of 3, 12, 24 and 72 h). *P < 0.05, **P < 0.01, and ***P < 0.001 compared with the normal group. ^#^*P* < 0.05, ^##^*P* < 0.01, and ^###^*P* < 0.001 compared with the HIBI group

Western blotting results suggested that CXCL1 expression levels were significantly increased at 12 (1.91 ± 0.32 vs. 1.30 ± 0.10), 24 (1.97 ± 0.31 vs. 0.76 ± 0.21), and 72 (2.70 ± 0.17 vs. 0.87 ± 0.25) h after hypoxic–ischemic induction (*P* < 0.05), and CXCR2 expression was significantly upregulated at 72 h (1.80 ± 0.40 vs. 0.88 ± 0.24, *P* < 0.05) in HIBI rats’ intestinal tissues compared to the control group (Fig. 4A). Similarly, CXCL1 and CXCR2 expression in the NEC + HIBI group was significantly elevated 12 h after hypoxic–ischemic induction compared to the control groups (*P* < 0.05). Compared with the HIBI group, the relative CXCL1 expression in the NEC + HIBI group increased significantly at 3 (1.82 ± 0.29 vs. 1.16 ± 0.17), 24 (3.17 ± 0.07 vs. 1.97 ± 0.31), and 72 (4.26 ± 0.25 vs. 2.70 ± 0.17) h (*P* < 0.01), while CXCR2 expression was notably upregulated only at 24 h (2.21 ± 0.14 vs. 1.09 ± 0.13, *P* < 0.001). Furthermore, in the brain tissues (Fig. 4B), CXCL1 levels in HIBI neonatal rats increased significantly 12 (3.13 ± 0.54 vs. 0.94 ± 0.14), 24 (4.35 ± 1.14 vs. 1.15 ± 0.39), and 72 (6.43 ± 1.06 vs. 1.01 ± 0.40) h after hypoxic–ischemic induction, and CXCR2 levels increased after 24 (1.63 ± 0.31 vs. 0.94 ± 0.33) and 72 (2.43 ± 0.58 vs. 1.19 ± 0.10) h (*P* < 0.05). CXCL1 and CXCR2 expression levels were also significantly higher in NEC + HIBI rats than in normal rats at all time points (*P* < 0.05). Compared with the HIBI group, CXCL1 expression in the NEC + HIBI group was significantly upregulated at all time points (*P* < 0.05), while that of CXCR2 was significantly increased 3 (1.52 ± 0.17 vs. 1.21 ± 0.10), 12 (2.36 ± 0.34 vs. 1.43 ± 0.24) and 24 (2.30 ± 0.11 vs. 1.63 ± 0.31) h after hypoxic–ischemic induction (*P* < 0.05). These results showed that CXCL1 and CXCR2 expression was upregulated to varying degrees in the intestinal and brain tissues of HIBI and NEC + HIBI neonatal rats, and that they may be involved in white matter injury via the gut-brain axis. Raw data of Fig. 4 including three replications are shown in Additional file 3: Figure S3 and Additional file 4: Figure S4.

## Discussion

With histopathological observation and protein level detection, this study observed brain tissue injury and increased CXCL1/CXCR2 expression levels in intestinal and brain tissues of NEC rats. After the successful construction of HIBI model, we found that from normal rats to HIBI rats and then to NEC + HIBI rats, the mortality of animals increased, accompanied by worsening brain and intestinal tissue damage, as well as increased CXCL1/CXCR2 expression levels, suggesting the occurrence of inflammation in intestinal and brain tissues.

NEC is a common complication in preterm infants. About 10% of preterm infants develop NEC, of which only 50% survive, and of those who survive, nearly half suffer from long-term neurological sequelae [26]. Biouss et al. experimentally showed that NEC severity in newborn rats is related to brain injury severity [27]. Martin et al. found that 24 month-old preterm infants with a history of NEC had an increased risk of neurodevelopmental dysfunction and microcephaly [28]. The present study also confirmed the increased possibility of periventricular white matter injury and decreased number of glial cells in neonates with NEC. As a potential regulatory mechanism of premature brain injury, the relationship between intestinal and nervous system diseases has been widely discussed in recent years, and the concept of the gut-brain axis has been derived.

The gut-brain axis is a communication system consisting of interactive and bidirectional neural, hormonal, and immune signals between the gut and brain, of which gut microbes are major contributors [29]. Ma et al. found changes in colon morphology and increased intestinal permeability in mice with traumatic brain injury [30]. Metagenomic sequencing has revealed that brain injury can affect the composition of the intestinal flora [31]. Studies have also shown that patients with central nervous system disorders, such as Parkinson’s disease, may experience gastrointestinal dysfunction before diagnosis [32]. These studies suggest that brain injury may affect intestinal injury through reverse signal transmission via the gut-brain axis. Our findings confirmed the above viewpoints and found that the intestinal tissues of neonatal rats were also damaged after HIBI. Likewise, intestinal and brain injuries were more severe in NEC + HIBI rats, suggesting that there may be a mechanism of gut–brain axis regulation in neonatal rats with hypoxic-ischemic brain injury. However, the molecular mechanisms that mediate the gut-brain axis have not been clearly studied. In addition to histopathological assessment, the present study also found increased mortality in HIBI and NEC + HIBI rats, suggesting that intestinal and brain injury may be one of the causes of mortality in neonatal rats. This finding is consistent with the conclusions of many studies based on humans. It is known that HIBI is a significant cause of mortality in the adults as well as in the neonates [33]. The mortality rate among neonates with severe NEC requiring surgery is estimated at 20–30% [34]. These studies further confirmed that we have been relatively successful in mimicking the disease environment of human NEC and HIBI in neonatal SD rats. However, the mortality of NEC combined with HIBI in neonates is not clear, and it was only reported that NEC occurs in 40% of hypoxic–ischemic neonates [35]. The results of this study suggested that the risk of death was about 1.4 times higher in NEC + HIBI rats than in HIBI rats, but epidemiological studies with a larger sample size based on human demographic statistics are still necessary to confirm the extrapolation of our results.

The gut is an important immune organ in the human body; gut epithelial cells, immune cells, and microorganisms maintain intestinal immune homeostasis through cytokine interaction [31]. When the intestinal immune system is imbalanced, pro-inflammatory cytokines may participate in brain inflammation through the gut-brain axis, thereby leading to brain damage [36]. CXCL1 is a pro-inflammatory chemokine that mediates immune cell migration after binding to CXCR2 and participates in biological effects, such as inflammation and immune responses. Previous reports have found that NEC disrupts intestinal immune homeostasis and increases CXCL1 expression in pathological intestinal tissue [37]. Meanwhile, CXCL1 induces peripheral neutrophil migration to the central nervous system by binding to CXCR2 [38]. Our study found that CXCL1 and CXCR2 expression was upregulated to varying degrees in the brain and intestinal tissues of NEC, HIBI, and NEC + HIBI neonatal rats, suggesting that CXCL1/CXCR2 may affect brain injury by delivering intestinal inflammatory mediators through the gut-brain axis. An intestinal flora-based study can explain our findings and indicate that changes in intestinal flora can induce NEC, allowing intestinal pro-inflammatory cytokines to penetrate the intestinal and blood–brain barriers, leading to systemic inflammatory responses and inflammatory brain injury [36]. Activated intestinal immune cells can also migrate to the site of brain injury and secrete pro-inflammatory cytokines to participate in the inflammatory response and aggravate brain injury [39]. Combined with our results, we conclude that intestinal immunity, inflammatory responses, and brain injury are interrelated, and that CXCL1/CXCR2 may play an important role in inflammatory transmission. These findings also give us some hints that the development of CXCL1 and CXCR2 inhibitors based on gut microbiota is expected to alleviate NEC symptoms, and reduce the risk of HIBI complication in infants with NEC, by blocking inflammatory mediators and inhibiting the migration of peripheral neutrophils to the central nervous system, but human-based in vitro and in vivo experiments are still urgently needed.

There are a few limitations associated with our study. First, the animal gender was not recorded in this study, resulting in the fact that the effect of sex variables on the results could not be assessed. Second, we could not analyze CXCL1 and CXCR2 expression in intestinal and brain tissues at the transcriptional level—doing so could have improved our study overall. Finally, the current study lacks an understanding of the immune response mechanisms involved in the CXCL1/CXCR2 pathway. In a follow-up study, we will conduct a blocking experiment on CXCR2, detect peripheral and central immune cell enrichment, and identify glial cell activation in rats to further explore CXCL1 regulatory mechanisms in the gut–brain axis.

## Conclusions

Through the construction of animal models, white matter injury was found in NEC rats, while intestinal inflammation was observed in HIBI rats, potentiating a bidirectional regulation mechanism of the gut–brain axis in intestinal and brain injuries. Furthermore, upregulation of CXCL1 and CXCR2 expression was detected in the intestinal and brain tissues of NEC, HIBI, and NEC + HIBI neonates, further indicating that CXCL1/CXCR2 may transmit inflammatory mediators through the gut-brain axis to influence brain injury.

## Supplementary Information

**Additional file 1: Figure S1.** WB raw data of Figure 2A.

**Additional file 2: Figure S2.** WB raw data of Figure 2B.

**Additional file 3: Figure S3.** WB raw data of Figure 4A.

**Additional file 4: Figure S4.** WB raw data of Figure 4B.

**Additional file 5: Table S1.** Pathological scoring criteria for intestinal tissue injury. **Table S2** Pathological scoring criteria for brain tissue injury.

## Data Availability

All data generated or analysed during this study are included in this published article (and its Additional files). The data used to support the findings of this study are available from the corresponding author upon request.
