# Supplementary figures and images for: CXCL1/CXCR2 is involved in white matter injury in neonatal rats via the gut–brain axis
Source: BMC Neurosci. 2022 Nov 19;23:67. doi: 10.1186/s12868-022-00749-1 (PMC9675237; doi:10.1186/s12868-022-00749-1)

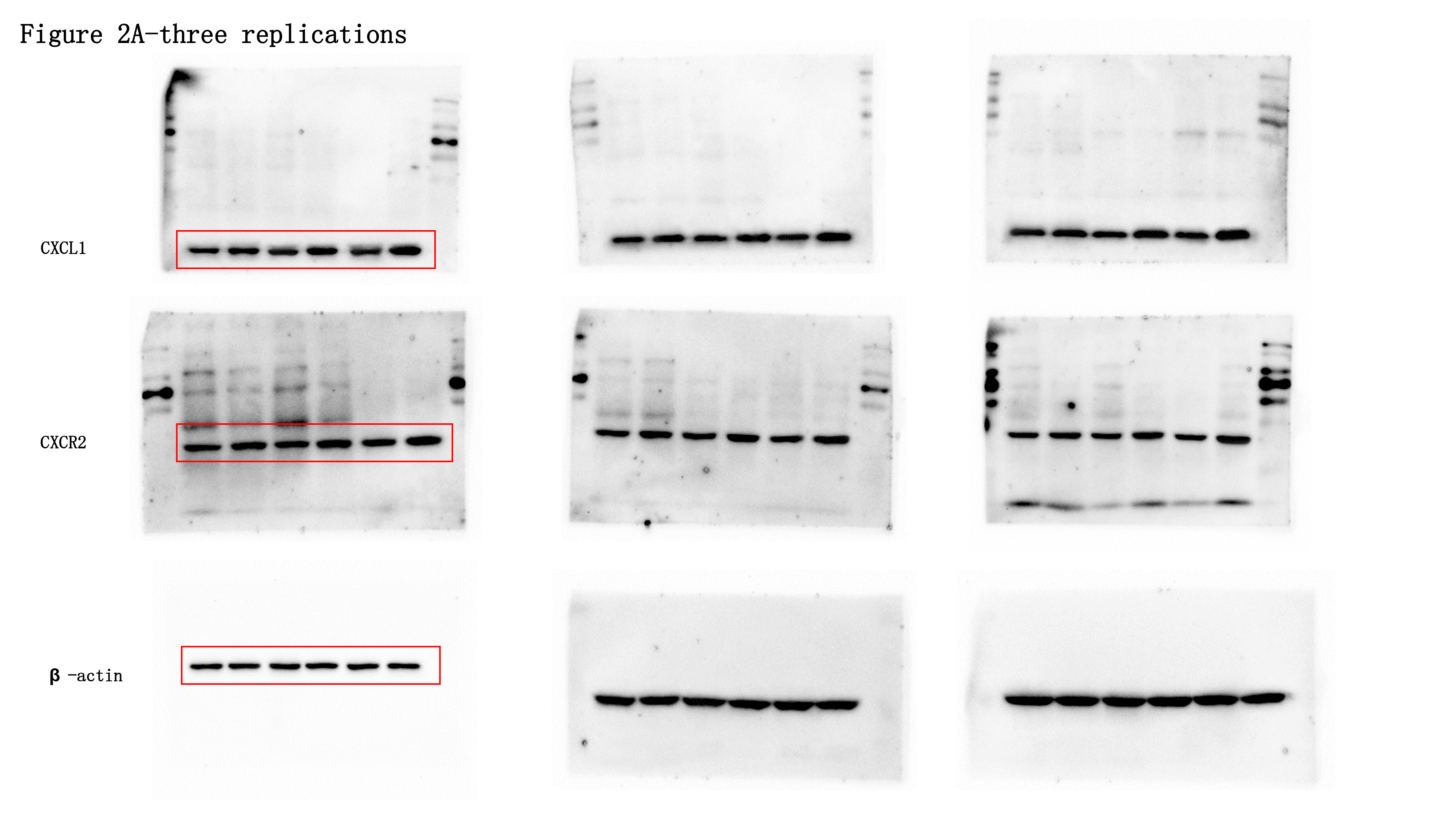

Supplement: Supplementary file 1 — Additional file 1: Figure S1. WB raw data of Figure 2A. [file 12868_2022_749_MOESM1_ESM.tif]

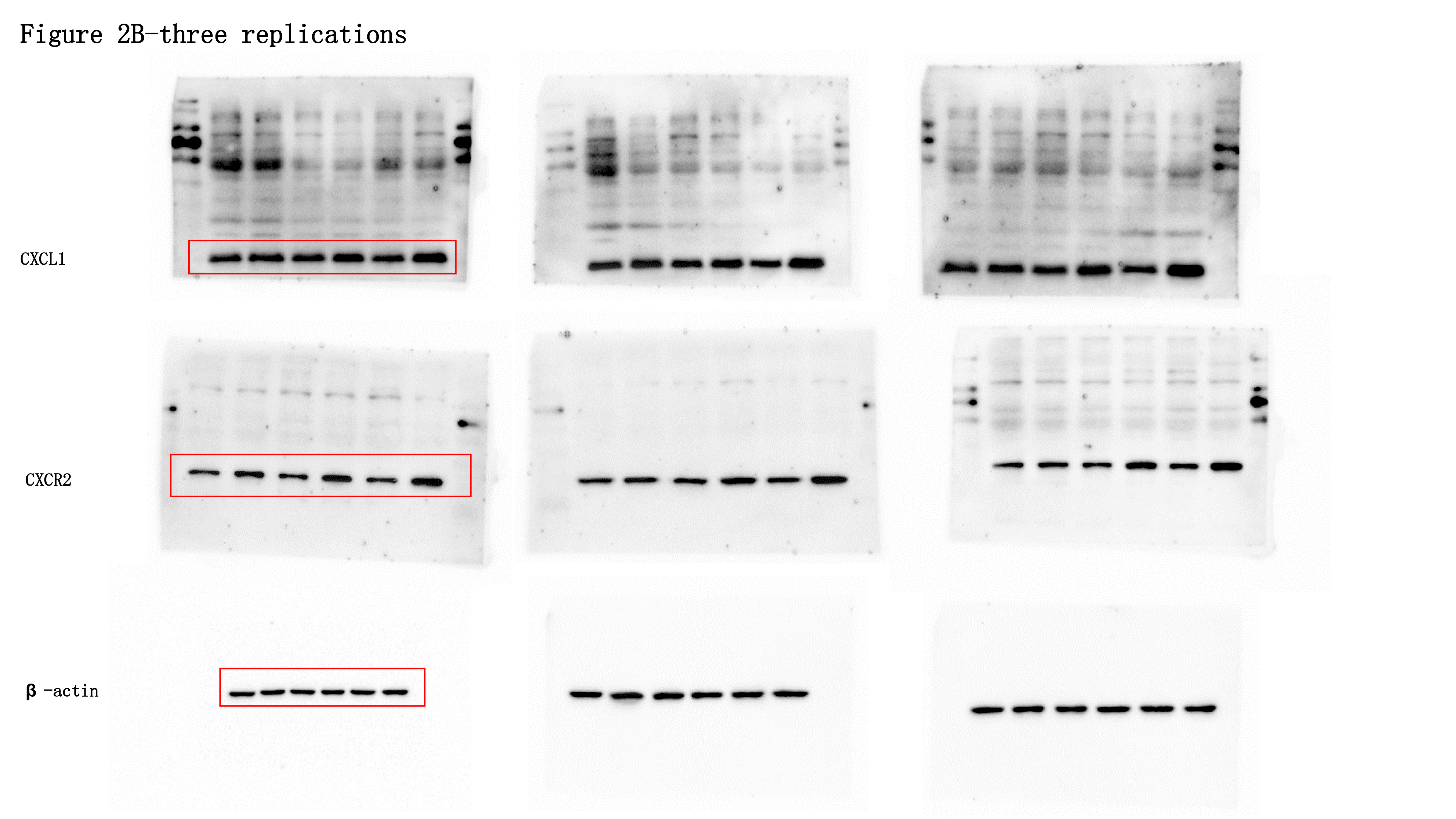

Supplement: Supplementary file 2 — Additional file 2: Figure S2. WB raw data of Figure 2B. [file 12868_2022_749_MOESM2_ESM.tif]

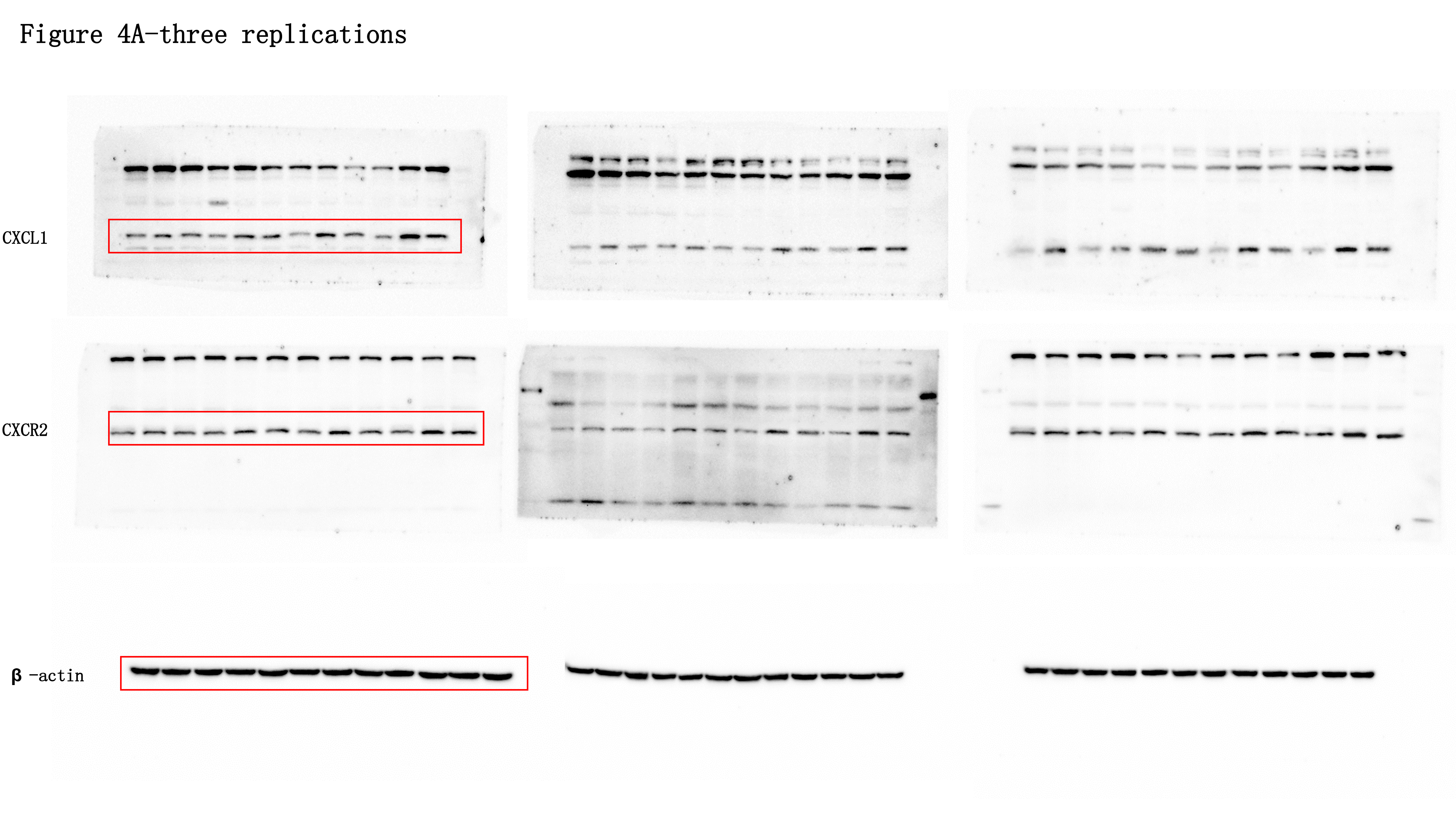

Supplement: Supplementary file 3 — Additional file 3: Figure S3. WB raw data of Figure 4A. [file 12868_2022_749_MOESM3_ESM.tif]

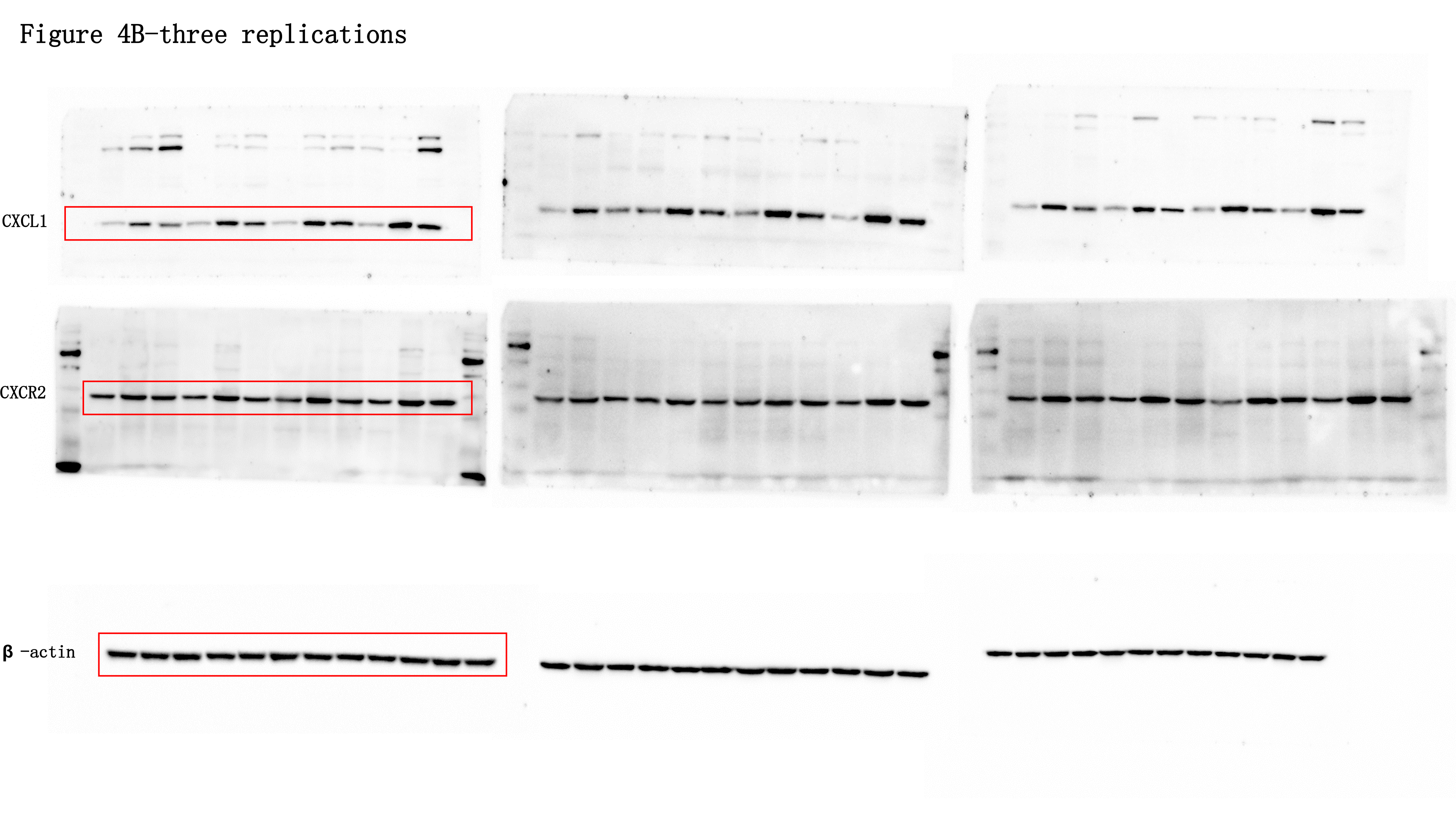

Supplement: Supplementary file 4 — Additional file 4: Figure S4. WB raw data of Figure 4B. [file 12868_2022_749_MOESM4_ESM.tif]
