# Supplementary material for: CXCL1/CXCR2 is involved in white matter injury in neonatal rats via the gut–brain axis
Source: BMC Neurosci. 2022 Nov 19;23:67. doi: 10.1186/s12868-022-00749-1 (PMC9675237; doi:10.1186/s12868-022-00749-1)
Supplement: Supplementary file 5 — Additional file 5: Table S1. Pathological scoring criteria for intestinal tissue injury. Table S2 Pathological scoring criteria for brain tissue injury. [file 12868_2022_749_MOESM5_ESM.docx]

**Additional file 5: Table S1 Pathological scoring criteria for intestinal tissue injury**

| **Score** | **Pathological features** |
| --- | --- |
| 0 | intact intestinal mucosa villi and normal intestinal structure |
| 1 | superficial epithelial cell sloughing |
| 2 | mid-villous necrosis |
| 3 | complete villous necrosis |
| 4 | transmural necrosis |

The histologic injury scores of intestinal tissues were defined by Ginzel et al. [23].

Neonatal rats with intestinal histopathological scores ≥ 2 were identified as the necrotizing enterocolitis-positive.

**Additional file 5: Table S2 Pathological scoring criteria for brain tissue injury**

| **Grade** | **Pathological features** |
| --- | --- |
| 0 | normal |
| 1 | Mildly abnormal (slightly loose white matter, and irregular arrangement of nerve fibers) |
| 2 | Severely abnormal (Severely loose white matter, disordered arrangement of nerve fibers, necrosis of coagulation, and the formation of cyst cavity) |

The histologic injury scores of brain tissues were defined by Uehara et al. [24].

Neonatal rats with brain histopathological scores ≥ 1 were considered the positive of hypoxic-ischemic brain injury.
